# Decent Work Among Hospital Nurses: Associations with Work Engagement, Burnout, and Job Crafting in a Cross-Sectional Study

**DOI:** 10.3390/nursrep16100355

**Published:** 2026-10-08

**Authors:** Yasue Yamazumi, Mina Sakurai, Rika Matsumoto

**Affiliations:** Faculty of Nursing, Kyoritsu Women’s University, Tokyo 101-0051, Japan

**Keywords:** decent work, hospital nurses, work engagement, burnout, job crafting, labour rights, nursing workforce

## Abstract

**Background/Objectives**: Decent Work (DW) has emerged as a comprehensive framework for understanding the quality of working conditions. However, evidence regarding DW among hospital nurses remains limited, particularly in Japanese hospitals with 200–399 beds. This study aimed to describe DW among hospital nurses working in hospitals with 200–399 beds in Japan and to examine its associations with work engagement, burnout, and job crafting. The five-factor structure of the Decent Work Scale for Hospital Nurses (DWS-N) was also re-examined in an independent sample. **Methods**: A cross-sectional, web-based survey was conducted among hospital nurses working in Japanese hospitals with 200–399 beds between 7 July and 2 August 2026. Of 348 responses received, 342 were included in the analysis. Participants completed the DWS-N, the Utrecht Work Engagement Scale, the Copenhagen Burnout Inventory, and the Job Crafting Scale. Descriptive statistics, confirmatory factor analysis (CFA), correlation analyses, and hierarchical multiple regression analyses were performed. The regression analyses examined associations between the five DWS-N dimensions and the study outcomes after adjustment for age and sex. **Results**: The mean DW score was 72.64 (*SD* = 13.97). The previously established five-factor DWS-N model showed acceptable approximate fit (CFI = 0.939, TLI = 0.929, RMSEA = 0.063). Total DW was positively correlated with work engagement (r = 0.550, *p* < 0.001) and all four job crafting dimensions (rs = 0.218–0.294, all *p* < 0.001), and negatively correlated with all three burnout dimensions (rs = −0.476 to −0.528, all *p* < 0.001). In adjusted regression analyses, Meaningful and Fulfilling Work was positively associated with work engagement and with three of the four job crafting dimensions and negatively associated with all three burnout dimensions. Supportive and Safe Working Environment was positively associated with work engagement and selected job crafting dimensions and negatively associated with client-related burnout. Appropriate Working Time and Work–Life Balance was negatively associated with personal and work-related burnout and positively associated with decreasing hindering job demands. Protection of Workers’ Rights showed negative adjusted associations with three job crafting dimensions. **Conclusions**: DW was associated with work engagement, burnout, and job crafting among hospital nurses working in Japanese hospitals with 200–399 beds. The dimension-specific findings suggest that different aspects of DW may show distinct associations with nurses’ psychological and proactive work-related outcomes. Given the cross-sectional design, these findings should be interpreted as associations rather than temporal or causal relationships. Longitudinal research is needed to examine the direction and temporal ordering of these relationships.

## 1. Introduction

Nurses constitute the largest group of healthcare professionals worldwide and play a central role in maintaining the accessibility, safety, and quality of healthcare services. Nevertheless, nursing work is characterised by substantial occupational demands, including staffing shortages, long working hours, shift work, emotional demands, and exposure to occupational risks [1,2,3]. These working conditions have been associated with adverse outcomes such as burnout, job dissatisfaction, turnover intention, and reduced quality of care [1,4,5,6]. Improving the conditions under which nurses work is therefore essential not only for nurses’ health and well-being but also for workforce retention and the sustainability of healthcare systems.

The concept of Decent Work (DW), proposed by the International Labour Organization (ILO), provides a comprehensive framework for considering the quality of work. DW encompasses opportunities for productive work that provides fair income, security in the workplace, social protection, rights at work, and opportunities for workers to express their concerns and participate in decisions affecting their working lives [7,8,9]. From a psychosocial perspective, DW also concerns how structural and institutional working conditions are experienced by individual workers [10,11,12]. This perspective is particularly relevant to nursing because nurses’ everyday work experiences are shaped not only by individual characteristics but also by organisational conditions such as staffing, workload, working time, occupational safety, remuneration, and the protection of workers’ rights.

To assess DW specifically in nursing, Yamazumi and Tanaka developed the Decent Work Scale for Hospital Nurses (DWS-N) [13]. The DWS-N operationalises DW as the experiential realisation of structural labour conditions and comprises 22 items across five dimensions: Meaningful and Fulfilling Work, Protection of Workers’ Rights, Supportive and Safe Working Environment, Appropriate Working Time and Work–Life Balance, and Adequate Compensation. The scale demonstrated satisfactory reliability and validity, and its five-factor structure was supported across independent samples [13]. The development of the DWS-N has therefore made it possible to assess the extent to which DW is actually experienced by hospital nurses rather than merely whether organisational provisions or labour protections formally exist. The original validation study also indicated the need for further examination of the scale in diverse healthcare settings and populations [13].

However, the extent to which DW is realised may differ according to the organisational context in which nurses work. Hospital size is one potentially important contextual factor in Japan. According to the 2024 Survey on Hospital Nursing conducted by the Japanese Nursing Association, the turnover rates of regularly employed nursing staff were 12.2% in hospitals with 200–299 beds and 11.5% in those with 300–399 beds, compared with 10.4% in both hospitals with 400–499 beds and those with 500 or more beds [14]. These differences indicate that nursing staff in hospitals with 200–399 beds constitute an important population in which to examine working conditions and understand the organisational factors relevant to nurse retention. Nevertheless, the extent to which DW is realised among nursing staff working in hospitals of this size has not been sufficiently clarified.

In addition to describing the level of DW, it is important to understand how DW relates to nurses’ occupational well-being and work behaviours. Work engagement is a positive, fulfilling, work-related state characterised by vigor, dedication, and absorption [15,16], whereas burnout represents a negative response to sustained occupational demands and has been associated with turnover among nurses [4,5,6]. Job crafting refers to self-initiated changes that employees make to their job demands and job resources to improve the fit between their work and their needs, abilities, and preferences [17,18,19]. In nursing, job crafting has been associated with work engagement and well-being, suggesting that proactive modification of work may contribute to positive occupational outcomes [20,21].

The Job Demands–Resources (JD-R) model provides a theoretical framework for understanding these relationships. According to the JD-R model, excessive job demands contribute to a health-impairment process leading to exhaustion and burnout, whereas job resources foster a motivational process that promotes work engagement and positive work-related outcomes [22,23]. Studies among nurses have similarly demonstrated that job demands and resources are associated with burnout, work engagement, and turnover-related outcomes [6,24]. From this perspective, DW may be understood as a comprehensive set of structural and experiential working conditions that may encompass or shape job resources relevant to sustainable nursing work. Conditions in which work is meaningful, workers’ rights are protected, the working environment is safe and supportive, working time is appropriately managed, and compensation is adequate may facilitate work engagement while reducing burnout.

Job crafting may also be relevant within this process. Within the JD-R framework, employees can proactively modify their job demands and resources through job crafting [18,19]. However, such proactive behaviour does not occur independently of the work environment. Employees may be better able to invest effort in reshaping their work when adequate organisational and psychological resources are available. Work engagement may therefore be closely related to the association between favourable working conditions and proactive work behaviour [19,20,21]. Examining DW, work engagement, burnout, and job crafting within an integrated framework may provide a more comprehensive understanding of how structural working conditions are associated with nurses’ psychological and behavioural responses to work.

Although previous studies have reported associations between decent work and work-related well-being, evidence among hospital nurses remains limited. In particular, little is known about how overall DW and its specific dimensions are associated with work engagement, burnout, and job crafting among hospital nurses in Japan. Examining these outcomes together may provide a more differentiated understanding of how specific aspects of nurses’ working conditions are related to both psychological well-being and proactive work behaviors.

Hospitals with 200–399 beds constitute an important segment of the Japanese healthcare system and were selected as the setting of interest in the present study. However, the findings should be interpreted as specific to nurses working in hospitals of this size rather than as evidence of differences between hospital-size categories.

Accordingly, the primary aims of this study were to (1) describe the level of DW among hospital nurses working in hospitals with 200–399 beds in Japan and (2) examine the associations of overall DW and its five dimensions with work engagement, burnout, and job crafting. As a secondary objective, the previously established five-factor structure of the DWS-N was examined in this independent sample.

## 2. Materials and Methods

### 2.1. Study Design

This study employed a cross-sectional, web-based survey design to examine Decent Work (DW) among hospital nurses and its relationships with work engagement, burnout, and job crafting.

### 2.2. Participants and Setting

The study targeted hospital nurses (registered nurses and midwives) working in Japanese hospitals with 200–399 beds.

Hospitals were randomly selected from a national database, and the nursing departments of 100 hospitals were invited to distribute the study materials to potentially eligible nurses. Study materials for 10 nurses were provided to each hospital, resulting in a planned recruitment pool of up to 1000 nurses. Institutional participation and distribution of the study materials were voluntary, and nursing departments were not required to report whether the materials were distributed or how many nurses actually received them. Because the web-based survey was anonymous and hospital identifiers were not collected, the number of hospitals represented among the respondents could not be determined. Consequently, hospital-level and individual-level response rates could not be calculated.

The recruitment target was determined a priori with the aim of obtaining approximately 300 valid responses, taking into account the planned CFA of the 22-item DWS-N and the sample-size considerations used in the original DWS-N validation study. Because sample-size requirements for CFA and multivariable analyses depend on model characteristics rather than a universal rule of thumb, this target was considered a pragmatic benchmark for the planned analyses [25]. The recruitment pool was therefore set substantially larger than the target analytical sample to account for the anticipated low response to the web-based survey and the possibility that not all invited hospitals would distribute the study materials.

Eligible participants were registered nurses and midwives who were employed on a full-time basis, engaged in night-shift work, and who had been employed at their current hospital for at least two years. Nurses who ordinarily participated in night-shift work but were temporarily working day shifts only at the time of the survey because of personal or family circumstances were considered eligible. Nurses in their first year of employment and nursing managers at the level of head nurse or above were excluded.

Hospitals with 200–399 beds were specifically selected because previous national data indicated that turnover rates among regularly employed hospital nurses in Japan were higher in hospitals of this size than in hospitals with 400 or more beds.

### 2.3. Data Collection

Data were collected between 7 July and 2 August 2026 using an anonymous, self-administered, web-based questionnaire created with Google Forms.

Research cooperation documents containing a QR code linked to the questionnaire were distributed through the nursing departments of participating hospitals. Participation was entirely voluntary. Hospital administrators were asked not to encourage or pressure hospital nurses to participate.

A total of 348 responses were received. One respondent who did not provide informed consent and five respondents who had worked at their current hospital for less than two years and therefore did not meet the eligibility criteria were excluded. Consequently, 342 responses were included in the final analysis.

### 2.4. Measures

#### 2.4.1. Decent Work

DW was assessed using the Decent Work Scale for Hospital Nurses (DWS-N), which was developed and validated specifically for hospital nurses in Japan [13]. The DWS-N consists of 22 items across five dimensions: Meaningful and Fulfilling Work, Protection of Workers’ Rights, Supportive and Safe Working Environment, Appropriate Working Time and Work–Life Balance, and Adequate Compensation [13].

Each item was answered using a five-category response format ranging from 1 (“strongly disagree”) to 5 (“strongly agree”).

The total DWS-N score was calculated by summing the 22 item scores, yielding a possible range of 22–110, with higher scores indicating higher levels of Decent Work. Dimension scores were calculated by summing the items corresponding to each of the five dimensions. The total score was used to represent overall DW, consistent with the original validation study [13], whereas the five dimension scores were used to examine dimension-specific associations with the study outcomes.

#### 2.4.2. Work Engagement

Work engagement was assessed using the Japanese version of the Utrecht Work Engagement Scale (UWES) [15,16]. The UWES consists of 17 items representing three dimensions: vigor, dedication, and absorption [15,16].

Each item was answered using a seven-category response format ranging from 0 (“never”) to 6 (“always”). Item scores were summed to calculate an overall work engagement score, with a possible range of 0–102; higher scores indicated greater work engagement. The total score was used in the present analyses because the Japanese validation study supported the use of an overall work engagement construct [16].

#### 2.4.3. Burnout

Burnout was assessed using the Japanese version of the Copenhagen Burnout Inventory (CBI) [26,27]. The CBI consists of 19 items assessing three dimensions: personal burnout (6 items), work-related burnout (7 items), and client-related burnout (6 items) [26,27].

Items were answered using response categories assessing either the frequency of burnout symptoms or the degree to which participants experienced burnout. Following the scoring procedure of the Japanese version of the CBI, item responses were converted to scores ranging from 0 to 100 in 25-point increments (0, 25, 50, 75, and 100), with the relevant item in the work-related burnout dimension reverse-scored. A mean score was then calculated separately for each of the three dimensions. Thus, scores for personal burnout, work-related burnout, and client-related burnout each ranged from 0 to 100, with higher scores indicating greater burnout. The three dimension scores were analyzed separately and were not combined into a single overall burnout score.

#### 2.4.4. Job Crafting

Job crafting was assessed using the Japanese version of the Job Crafting Scale [17,28]. The scale consists of 21 items assessing four dimensions: increasing structural job resources, decreasing hindering job demands, increasing social job resources, and increasing challenging job demands [17,28].

Items were answered using a five-category response format ranging from 1 (“never”) to 5 (“very often”). The possible score ranges were 5–25 for increasing structural job resources, 6–30 for decreasing hindering job demands, 5–25 for increasing social job resources, and 5–25 for increasing challenging job demands. The four dimension scores were analyzed separately rather than combined into a single overall job crafting score, consistent with the multidimensional structure of the Japanese version [28]. Higher scores indicated greater levels of the corresponding job crafting behavior.

### 2.5. Statistical Analysis

Descriptive statistics were calculated for participant characteristics and all study variables. Internal consistency was evaluated using Cronbach’s α coefficients.

Confirmatory factor analysis (CFA) was conducted to examine the previously established five-factor structure of the DWS-N in this independent sample. The five-category item responses were treated as continuous variables, and maximum likelihood estimation was used. Multivariate normality was assessed using multivariate kurtosis. Because evidence of multivariate non-normality was observed, a Bollen–Stine bootstrap with 5000 resamples was additionally performed to assess model fit under non-normality. The CFA model was specified to correspond to the five-factor measurement model established in Phase 3 of the original DWS-N validation study [13], including the residual covariances retained in that model. Each item was specified to load only on its designated factor, the five latent factors were allowed to correlate, and the residual covariances retained in the original Phase 3 model were specified a priori. No additional residual covariances or other post hoc model modifications were introduced in the present study based on modification indices. Model fit was evaluated using the chi-square statistic (*χ*^2^), the chi-square/degrees-of-freedom ratio (*χ*^2^/df), the goodness-of-fit index (GFI), the adjusted goodness-of-fit index (AGFI), the comparative fit index (CFI), the Tucker–Lewis index (TLI), the root mean square residual (RMR), and the root mean square error of approximation (RMSEA) with its 90% confidence interval. Standardized factor loadings were also examined.

Pearson’s correlation coefficients were calculated to examine associations among the total DW score, the five DWS-N dimension scores, the overall work engagement score, the three burnout dimension scores, and the four job crafting dimension scores.

Hierarchical multiple regression analyses were conducted to examine the associations of the five DWS-N dimensions with work engagement, the three burnout dimensions, and the four job crafting dimensions. Age and sex were entered in Step 1, followed by the five DWS-N dimension scores in Step 2. Sex was dummy coded as female = 1 and male = 0; responses of “Other” and “Prefer not to answer” were treated as missing for the regression analyses. Multicollinearity was assessed using variance inflation factors (VIFs). Unstandardized regression coefficients (B), standard errors (SEs), standardized regression coefficients (*β*), 95% confidence intervals (CIs) for B, and VIFs were obtained. Model performance was evaluated using *R*^2^, adjusted *R*^2^, the change in *R*^2^ (Δ*R*^2^), and the F test for the change in *R*^2^.

Demographic and occupational characteristic items were optional; therefore, denominators for these variables varied according to the number of responses obtained. In contrast, all items of the DWS-N, UWES, CBI, and Job Crafting Scale were mandatory in the web-based questionnaire, and there were no missing data for these scale variables. Accordingly, descriptive statistics, internal consistency analyses, CFA, and correlation analyses were based on the full analytical sample (*n* = 342). Hierarchical multiple regression analyses were conducted using complete cases for age and sex (*n* = 316).

All statistical analyses were conducted using IBM SPSS Statistics version 30 (IBM Corp., Armonk, NY, USA) and IBM SPSS Amos version 28 (IBM Corp., Armonk, NY, USA). Statistical significance was set at *p* < 0.05.

This manuscript was prepared with reference to the Strengthening the Reporting of Observational Studies in Epidemiology (STROBE) Statement for cross-sectional studies [29].

### 2.6. Ethical Considerations

This study was conducted in accordance with the principles of the Declaration of Helsinki.

Ethical approval was obtained from the Research Ethics Committee of Kyoritsu Women’s University and Kyoritsu Women’s Junior College (Approval No. KWU-IRBA#26010; approved on 15 June 2026).

Before accessing the questionnaire, participants were provided with written information describing the study purpose, procedures, voluntary nature of participation, protection of confidentiality, and their right to decline participation without disadvantage.

Informed consent was obtained electronically through a consent checkbox included in the web-based questionnaire. Because the survey was conducted anonymously, submitted responses could not be withdrawn.

### 2.7. Use of Generative Artificial Intelligence

During the preparation of this manuscript, the author used a generative artificial intelligence tool (ChatGPT, OpenAI, GPT-4 series) for English-language editing and refinement. The author reviewed and edited all outputs and takes full responsibility for the accuracy and integrity of the manuscript.

## 3. Results

### 3.1. Participant Characteristics

A total of 348 responses were received. One respondent who did not provide informed consent and five respondents who had worked at their current hospital for less than two years and therefore did not meet the eligibility criteria were excluded. Consequently, 342 responses were included in the final analysis.

The mean age of the participants was 39.37 years (*SD* = 10.34, *n* = 326). The mean duration of nursing experience was 16.25 years (*SD* = 10.00, *n* = 320), and the mean duration of employment at the current institution was 11.88 years (*SD* = 8.50, *n* = 324).

Detailed demographic and occupational characteristics of the participants are presented in Table 1.

### 3.2. Descriptive Statistics and Internal Consistency of the Measures

The mean total DW score was 72.64 (*SD* = 13.97). Among the five DWS-N dimensions, the mean scores were 17.52 (*SD* = 3.70) for Meaningful and Fulfilling Work, 17.33 (*SD* = 4.10) for Protection of Workers’ Rights, 20.88 (*SD* = 4.60) for Supportive and Safe Working Environment, 9.09 (*SD* = 2.81) for Appropriate Working Time and Work–Life Balance, and 7.82 (*SD* = 2.96) for Adequate Compensation.

The mean work engagement score was 41.85 (*SD* = 20.17). The mean burnout scores were 52.67 (*SD* = 21.46) for personal burnout, 52.94 (*SD* = 20.30) for work-related burnout, and 51.22 (*SD* = 24.39) for client-related burnout. The mean scores for the four job crafting dimensions were 14.31 (*SD* = 4.11) for increasing structural job resources, 16.13 (*SD* = 4.74) for decreasing hindering job demands, 11.94 (*SD* = 4.01) for increasing social job resources, and 11.89 (*SD* = 4.15) for increasing challenging job demands (Table 2).

### 3.3. Confirmatory Factor Analysis of the DWS-N

CFA was conducted to examine the five-factor measurement model established in Phase 3 of the original DWS-N validation study in the present independent sample. The model included the residual covariances retained in the original Phase 3 model, and no additional post hoc modifications were made in the present analysis. Assessment of multivariate normality indicated substantial multivariate non-normality (multivariate kurtosis = 92.457, critical ratio = 26.308). Therefore, a Bollen–Stine bootstrap with 5000 resamples was performed; all 5000 bootstrap samples were successfully obtained. The Bollen–Stine bootstrap test was significant (*p* < 0.001).

**Figure 1:**
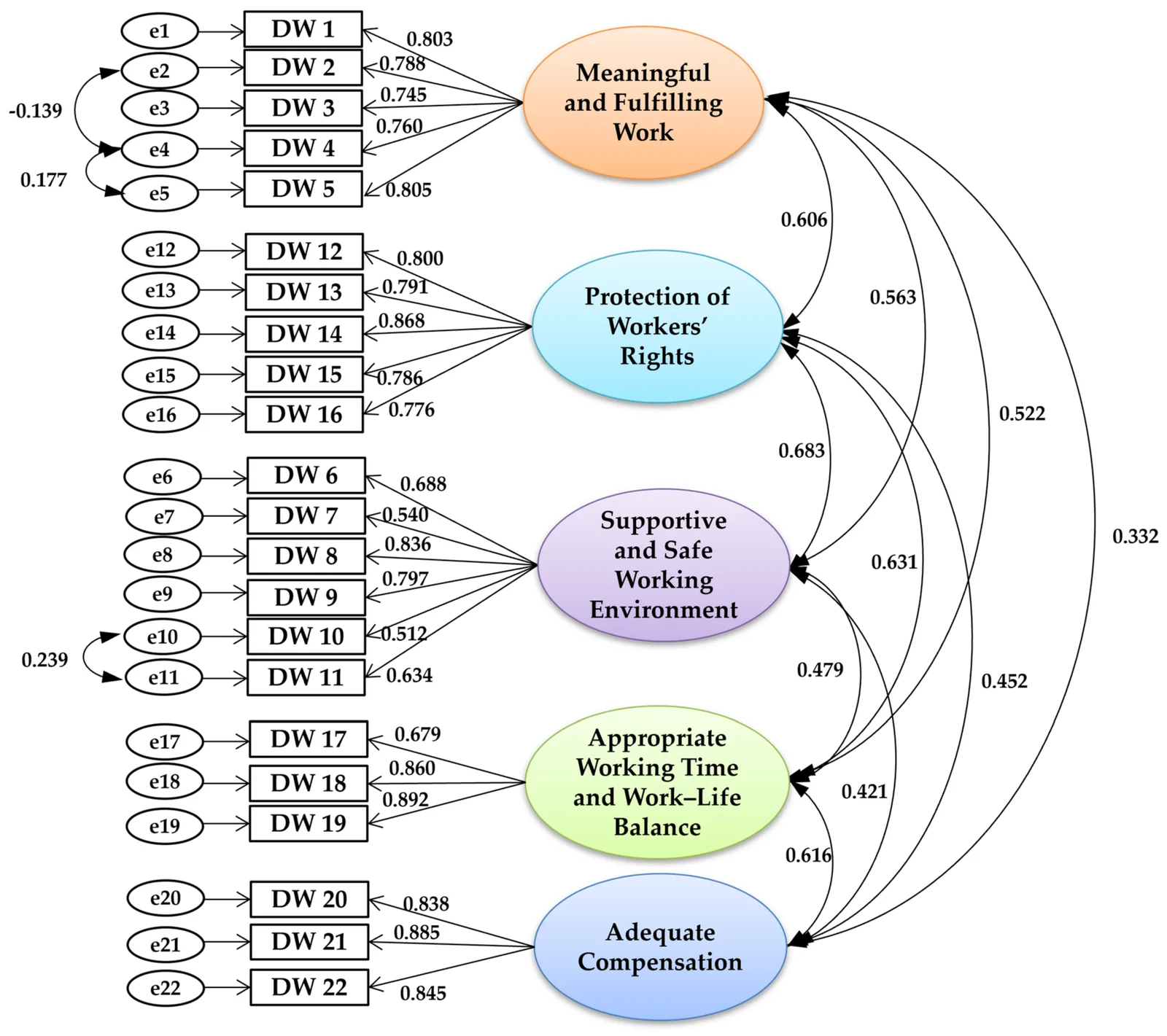
Confirmatory factor analysis of the Decent Work Scale for Hospital Nurses (DWS-N) (*N* = 342). Note. The five-factor model showed acceptable approximate fit based on conventional fit indices (*χ*^2^ = 469.964, *df* = 198, χ^2^/*df* = 2.374, CFI = 0.939, TLI = 0.929, GFI = 0.891, AGFI = 0.861, RMR = 0.064, RMSEA = 0.063, 90% CI [0.056, 0.071]). Given the observed multivariate non-normality, model fit was additionally evaluated using a Bollen–Stine bootstrap with 5000 resamples (*p* < 0.001).

The previously established five-factor model showed acceptable fit based on the approximate fit indices: *χ*^2^(198) = 469.964, *p* < 0.001, *χ*^2^/df = 2.374, GFI = 0.891, AGFI = 0.861, CFI = 0.939, TLI = 0.929, and RMSEA = 0.063 (90% CI [0.056, 0.071]). Standardized factor loadings ranged from 0.51 to 0.91. These findings provided reasonable support for the previously established five-factor structure of the DWS-N in the present sample, although the significant Bollen–Stine test indicated that exact model fit was not supported. The standardized estimates for the five-factor CFA model are presented in Figure 1.

### 3.4. Correlations Among Decent Work, Work Engagement, Burnout, and Job Crafting

Pearson’s correlation coefficients among the study variables are presented in Table 3. Total DW was positively correlated with overall work engagement (*r* = 0.550, *p* < 0.001). Total DW was negatively correlated with personal burnout (*r* = −0.509, *p* < 0.001), work-related burnout (*r* = −0.528, *p* < 0.001), and client-related burnout (*r* = −0.476, *p* < 0.001). Total DW was also positively correlated with all four job crafting dimensions: increasing structural job resources (*r* = 0.294, *p* < 0.001), decreasing hindering job demands (*r* = 0.232, *p* < 0.001), increasing social job resources (*r* = 0.218, *p* < 0.001), and increasing challenging job demands (*r* = 0.272, *p* < 0.001).

Overall work engagement was negatively correlated with all three burnout dimensions (*rs* = −0.381 to −0.433, all *p* < 0.001) and positively correlated with all four job crafting dimensions (*rs* = 0.167 to 0.496, all *p* ≤ 0.002). Correlations among the five DWS-N dimensions and the outcome variables are shown in Table 3.

### 3.5. Associations Between Decent Work Dimensions and Study Outcomes

Hierarchical multiple regression analyses were conducted to examine the associations of the five DWS-N dimensions with work engagement, the three burnout dimensions, and the four job crafting dimensions, after adjustment for age and sex (Table 4, Table 5 and Table 6).

For work engagement, age and sex entered in Step 1 explained 0.9% of the variance (*R*^2^ = 0.009, *p* = 0.230). Adding the five DWS-N dimensions in Step 2 explained an additional 36.9% of the variance (Δ*R*^2^ = 0.369, *p* < 0.001), resulting in a final *R*^2^ of 0.379 (adjusted *R*^2^ = 0.364). In the final model, Meaningful and Fulfilling Work (*β* = 0.435, *p* < 0.001) and Supportive and Safe Working Environment (*β* = 0.147, *p* = 0.017) were positively associated with work engagement.

For the burnout dimensions, addition of the five DWS-N dimensions in Step 2 significantly increased the explained variance in personal burnout (Δ*R*^2^ = 0.339, *p* < 0.001), work-related burnout (Δ*R*^2^ = 0.311, *p* < 0.001), and client-related burnout (Δ*R*^2^ = 0.234, *p* < 0.001). The final models explained 36.1%, 33.8%, and 25.4% of the variance, respectively. Meaningful and Fulfilling Work was negatively associated with all three burnout dimensions (*β* = −0.293, −0.256, and −0.246, respectively; all *p* < 0.001). Appropriate Working Time and Work–Life Balance was negatively associated with personal burnout (*β* = −0.393, *p* < 0.001) and work-related burnout (*β* = −0.298, *p* < 0.001), whereas Supportive and Safe Working Environment was negatively associated with client-related burnout only (*β* = −0.179, *p* = 0.008).

For the job crafting dimensions, addition of the five DWS-N dimensions in Step 2 significantly increased the explained variance in increasing structural job resources (Δ*R*^2^ = 0.176, *p* < 0.001), decreasing hindering job demands (Δ*R*^2^ = 0.109, *p* < 0.001), increasing social job resources (Δ*R*^2^ = 0.066, *p* < 0.001), and increasing challenging job demands (Δ*R*^2^ = 0.127, *p* < 0.001). The final models explained 18.5%, 13.5%, 10.7%, and 13.4% of the variance, respectively. Meaningful and Fulfilling Work was positively associated with increasing structural job resources (*β* = 0.400, *p* < 0.001), decreasing hindering job demands (*β* = 0.167, *p* = 0.015), and increasing challenging job demands (*β* = 0.332, *p* < 0.001). Protection of Workers’ Rights was negatively associated with increasing structural job resources (*β* = −0.202, *p* = 0.008), decreasing hindering job demands (*β* = −0.263, *p* < 0.001), and increasing social job resources (*β* = −0.173, *p* = 0.031). Supportive and Safe Working Environment was positively associated with decreasing hindering job demands (*β* = 0.146, *p* = 0.042) and increasing social job resources (*β* = 0.158, *p* = 0.031), while Appropriate Working Time and Work–Life Balance was positively associated with decreasing hindering job demands (*β* = 0.230, *p* = 0.003).

VIFs in the final models ranged from 1.010 to 2.181, indicating no evidence of problematic multicollinearity.

## 4. Discussion

### 4.1. Decent Work Among Hospital Nurses Working in Hospitals with 200–399 Beds

This study examined DW and its associations with work engagement, burnout, and job crafting among hospital nurses working in Japanese hospitals with 200–399 beds. The mean total DW score was 72.64 (*SD* = 13.97). Hospitals with 200–399 beds were selected as the setting of interest because national data indicate somewhat higher turnover rates among regularly employed nursing staff in hospitals of this size than in hospitals with 400 or more beds [14]. However, because the present study included only hospitals with 200–399 beds and did not directly assess turnover, the findings should not be interpreted as evidence that DW differs according to hospital size or that DW accounts for differences in turnover rates.

The importance of examining DW as a comprehensive construct is supported by recent evidence in nursing and care work. A mixed-method systematic review by Hult et al. found that decent work and precarious work represent contrasting dimensions of employment quality and that decent work was associated with better physical and mental health, greater empowerment, and lower turnover among nursing and care workers [30]. These findings support the relevance of examining not only isolated working conditions but also the combined experience of meaningful work, workers’ rights, the work environment, working time, and compensation.

The present study therefore provides an empirical description of DW in an independent sample of hospital nurses working in hospitals with 200–399 beds in Japan. However, because hospital participation and distribution of study materials were voluntary and the number of hospitals represented among respondents could not be determined, the observed DW level should not be regarded as representative of all hospital nurses working in hospitals of this size in Japan. Direct comparisons of DW across hospital-size categories will require studies specifically designed for this purpose.

### 4.2. Five-Factor Structure of the DWS-N in an Independent Sample

The previously established five-factor structure of the DWS-N showed acceptable approximate fit in the present independent sample (CFI = 0.939, TLI = 0.929, and RMSEA = 0.063). These findings provide additional support for the structural validity of the DWS-N beyond the samples used in its original development and validation [13].

The model fit in the present study was somewhat better than that obtained in Study 3 of the original validation study, in which the CFI was 0.917 and the RMSEA was 0.070 [13]. However, rather than indicating superior structural validity, the present findings provide further evidence that the same five-factor structure shows acceptable approximate fit in an independent sample of hospital nurses.

This additional evidence is particularly relevant because DW is inherently multidimensional. The five DWS-N dimensions—Meaningful and Fulfilling Work, Protection of Workers’ Rights, Supportive and Safe Working Environment, Appropriate Working Time and Work–Life Balance, and Adequate Compensation—represent distinct but related aspects of how structural labour conditions are experienced in nursing practice [13]. The support for this structure in the present sample strengthens the rationale for using the DWS-N to examine DW across different hospital settings.

### 4.3. Associations of Decent Work with Work Engagement, Burnout, and Job Crafting

DW was positively correlated with work engagement and all four dimensions of job crafting and negatively correlated with all three dimensions of burnout. These findings are consistent with the JD-R framework, in which job resources promote motivational processes and work engagement, whereas excessive demands and insufficient resources contribute to health impairment and burnout [22,23].

Recent nursing evidence supports this interpretation. A 2025 systematic review of nursing practice environments found that more favourable professional practice environments were consistently associated with higher work engagement and lower burnout among nurses [31]. Thus, the present findings are consistent with a broader body of evidence showing that nurses’ occupational responses are closely related to the environments in which they work.

However, DW is broader than the nursing practice environment alone. It includes labour rights, working time, compensation, meaningful work, and the extent to which supportive and safe working conditions are actually experienced. The present findings therefore suggest that DW may provide an integrative framework for examining several structural and experiential conditions simultaneously, rather than treating each working condition as an isolated exposure. This interpretation is also consistent with the recent systematic review of decent and precarious work among nursing and care workers, which emphasized employment quality as a multidimensional phenomenon with implications for health, empowerment, and turnover [30].

### 4.4. Differential Associations of the Five Dimensions of Decent Work

The multiple regression analyses indicated that the five dimensions of DW were not uniformly associated with work engagement, the three burnout dimensions, and the four job crafting dimensions. Meaningful and Fulfilling Work and Supportive and Safe Working Environment were associated with higher work engagement after adjustment for age and sex. Meaningful and Fulfilling Work was negatively associated with all three burnout dimensions, Appropriate Working Time and Work–Life Balance was negatively associated with personal and work-related burnout, and Supportive and Safe Working Environment was negatively associated with client-related burnout. For job crafting, the associations also differed across dimensions: Meaningful and Fulfilling Work was positively associated with increasing structural job resources, decreasing hindering job demands, and increasing challenging job demands, whereas Protection of Workers’ Rights showed negative associations with increasing structural job resources, decreasing hindering job demands, and increasing social job resources. Supportive and Safe Working Environment was positively associated with decreasing hindering job demands and increasing social job resources, while Appropriate Working Time and Work–Life Balance was positively associated with decreasing hindering job demands.

Meaningful and Fulfilling Work showed the most consistent pattern of associations across the study outcomes, being associated with higher work engagement, lower scores on all three burnout dimensions, and three of the four job crafting dimensions. This finding is consistent with recent literature emphasizing the importance of meaning in nurses’ work. A 2024 scoping review of hospital nurses identified recognition, trust, autonomy, relationships with patients, opportunities for learning and development, moral integrity, and involvement in work as important components of how nurses experience meaning in their work [32]. These attributes overlap conceptually with the DWS-N dimension of Meaningful and Fulfilling Work and provide a plausible basis for its associations with motivational, health-related, and proactive work-related outcomes.

Supportive and Safe Working Environment was associated with higher work engagement and lower client-related burnout. This finding is consistent with recent systematic evidence indicating that favourable nursing practice environments are associated with greater engagement and lower burnout [31]. However, in the present study, Supportive and Safe Working Environment was not significantly associated with personal or work-related burnout after simultaneous adjustment for the other DW dimensions. This pattern suggests that the associations between supportive and safe working conditions and burnout may differ across burnout dimensions.

Appropriate Working Time and Work–Life Balance was associated with lower personal and work-related burnout and was also positively associated with decreasing hindering job demands. The relationships with burnout are consistent with contemporary evidence concerning nurses’ working hours and scheduling. Reviews of nursing work have repeatedly identified extended shifts, workload, and difficulties balancing professional and personal demands as relevant to fatigue and burnout, and a 2024 critical review of 12-h shifts similarly reported adverse associations with fatigue, burnout, job satisfaction, and intention to leave, although the evidence was heterogeneous [33].

The positive association between Appropriate Working Time and Work–Life Balance and decreasing hindering job demands is also noteworthy. Job crafting requires employees to actively modify job demands and resources [17,18,19]. Sufficient time and recovery opportunities may leave nurses with greater capacity to adjust aspects of their work that they perceive as burdensome or obstructive. This interpretation should remain cautious because the present cross-sectional design cannot establish directionality. Nevertheless, recent nursing research has linked job crafting with retention-related outcomes, and a 2024 study found that specific job-crafting behaviours were associated with nurses’ intention to stay in their hospitals and profession [34].

An unexpected finding was the negative associations between Protection of Workers’ Rights and several job crafting dimensions. Protection of Workers’ Rights was positively correlated with these job crafting dimensions in the bivariate analyses but showed negative associations with increasing structural job resources, decreasing hindering job demands, and increasing social job resources after simultaneous adjustment for the other DW dimensions. This reversal in the direction of the associations suggests that the findings may partly reflect shared variance among the dimensions of DW or suppression effects rather than straightforward negative relationships between workers’ rights and proactive work behaviour. Because the five DW dimensions were moderately intercorrelated, the regression coefficients represent the association of each dimension with job crafting after accounting for variance shared with the other DW dimensions. Thus, the negative coefficients for Protection of Workers’ Rights may reflect its unique residual variance after this shared variance was statistically controlled, rather than indicating that stronger protection of workers’ rights is itself associated with less job crafting. Accordingly, these results should be interpreted cautiously and require further investigation in other samples.

In contrast, Adequate Compensation was not significantly associated with work engagement, any of the three burnout dimensions, or any of the four job crafting dimensions after the five DW dimensions were considered simultaneously. This finding should not be interpreted as indicating that compensation is an unimportant component of DW. Rather, compensation may be related to other outcomes, such as perceived fairness, intention to leave, or employment security, which were not examined in the present study.

### 4.5. Implications for Nursing Management and Workforce Policy

The findings have practical implications for nursing management because they suggest that improving DW requires more than interventions directed at individual coping or resilience. Recent reviews of burnout interventions among nurses have found beneficial effects from a range of interventions, but organisational and workplace conditions remain central to sustainable improvement [35,36].

In particular, Meaningful and Fulfilling Work, Supportive and Safe Working Environment, and Appropriate Working Time and Work–Life Balance showed associations with multiple study outcomes, suggesting that these dimensions may warrant close organisational attention. However, these findings should not be interpreted as indicating that Protection of Workers’ Rights or Adequate Compensation is less important. Rather, the DWS-N can be used to assess all five dimensions and identify which aspects of DW are less fully realised within a particular organisation.

This broader perspective is consistent with recent evidence on employment quality in nursing and care work. Hult et al. concluded that decent work is associated with positive health and empowerment outcomes and lower turnover, while emphasizing the need for sustainable employment policies and attractive career prospects [30]. Thus, DW may offer nursing managers and policymakers a framework for considering individual occupational experiences in relation to organisational labour conditions. However, workforce retention was not assessed in the present study, and its relationship with DW should be examined directly in future longitudinal research.

### 4.6. Limitations and Future Directions

Several limitations should be considered. First, the cross-sectional design precludes causal inference and does not establish the temporal ordering of the observed associations. Longitudinal data are needed to determine whether DW predicts subsequent changes in work engagement, burnout, and job crafting, and whether reciprocal effects occur.

Second, because hospital identifiers were not collected in the anonymous survey, potential clustering of nurses within hospitals could not be accounted for in the analyses, and multilevel modeling could not be performed. If responses were correlated within hospitals, the standard errors may have been underestimated. In addition, because nursing departments were not required to report whether the study materials were distributed or how many nurses received them, the number of hospitals represented in the sample and hospital- and individual-level response rates could not be determined. This limits the assessment of potential selection and nonresponse bias and the representativeness of the sample. Accordingly, the precision of the estimated associations and their statistical significance should be interpreted with caution.

Third, the study was restricted to hospital nurses working in hospitals with 200–399 beds. The findings therefore should not be generalized directly to larger hospitals or other healthcare settings. A direct comparison between hospital-size categories is needed before concluding that the level or effects of DW differ according to hospital size.

Fourth, all variables were assessed using self-report measures collected through a web-based survey. Common-method and response biases therefore remain possible. Future studies could incorporate organisational indicators, staffing data, working-hour records, turnover data, or repeated measurements to complement self-reported experiences.

Fifth, the five DWS-N dimensions were examined simultaneously in the regression analyses. Therefore, the unexpected negative association between Protection of Workers’ Rights and several job crafting dimensions and the absence of significant associations for Adequate Compensation should be interpreted cautiously. These findings may reflect overlapping variance among the DW dimensions or relationships with occupational outcomes not examined in the present study.

Future research should examine DW longitudinally and compare nurses working in hospitals of different sizes. Such studies would permit evaluation of whether differences in DW precede changes in work engagement, burnout, and job crafting, as well as subsequent employment continuity. They would also help clarify whether the relative importance of the five DW dimensions differs according to organisational context.

Overall, the present findings support the relevance of DW as a multidimensional framework for understanding hospital nurses’ working conditions. Rather than positioning burnout, engagement, or job crafting solely as individual-level phenomena, the findings indicate that these occupational outcomes should also be considered in relation to the structural and experiential conditions under which nurses work.

## 5. Conclusions

This study examined Decent Work (DW) among hospital nurses working in Japanese hospitals with 200–399 beds and explored its relationships with work engagement, burnout, and job crafting.

The previously established five-factor structure of the Decent Work Scale for Hospital Nurses (DWS-N) showed acceptable approximate fit in an independent sample, providing additional support for its structural validity.

DW was positively associated with work engagement and all four job crafting dimensions and negatively associated with all three burnout dimensions. Furthermore, different dimensions of DW demonstrated distinct patterns of association with occupational outcomes. Meaningful and Fulfilling Work showed the most consistent pattern of associations across the outcomes, including higher work engagement, lower scores on all three burnout dimensions, and higher scores on three job crafting dimensions. Supportive and Safe Working Environment was associated with higher work engagement and lower client-related burnout, whereas Appropriate Working Time and Work–Life Balance was associated with lower personal and work-related burnout and with job crafting related to reducing hindering job demands.

Overall, the present findings support the usefulness of DW as a multidimensional framework for understanding hospital nurses’ working conditions. Improving nurses’ working conditions should involve not only reducing occupational risks but also promoting meaningful work experiences and supporting appropriate working time and work–life balance. This study provides a foundation for future comparative and longitudinal research examining temporal relationships between working conditions and nurses’ occupational outcomes, including subsequent employment continuity.

## Figures and Tables

**Table 1:** Demographic and Occupational Characteristics of the Participants (*N* = 342).

Variable		Category	*n* (%) or Mean ± *SD*
Age (years)	*n* = 326		39.37 ± 10.34
Years of nursing experience	*n* = 320		16.25 ± 10.00
Years of tenure	*n* = 324		11.88 ± 8.50
Sex	*n* = 334	Female	286 (85.6)
		Male	41 (12.3)
		Other	2 (0.6)
		Prefer not to answer	5 (1.5)
Partner status	*n* = 335	Yes	209 (62.4)
		No	126 (37.6)
Children	*n* = 338	Yes	182 (53.8)
		No	156 (46.2)
Experience of providing care for family members	*n* = 339	Yes	99 (29.2)
		No	240 (70.8)
Certification	*n* = 337	Yes	40 (11.9)
		No	297 (88.1)
Work schedule	*n* = 340	Day shift only (temporary)	35 (10.3)
		Two-shift system	237 (69.7)
		Three-shift system	35 (10.3)
		Day–night duty system	5 (1.5)
		On-call duty system	2 (0.6)
		Irregular schedule	26 (7.6)
Workplace childcare availability	*n* = 340	Yes	252 (74.1)
		No	88 (25.9)
Paid leave availability	*n* = 340	Available	251 (73.8)
		Not available	89 (26.2)

Values are presented as *n* (%). Note. Demographic and occupational characteristic items were optional; therefore, denominators vary across variables. Day-night duty system refers to a work schedule consisting of regular daytime duties combined with overnight duty periods. “Day shift only (temporary)” refers to nurses who ordinarily participate in night-shift work but were temporarily working day shifts only at the time of the survey because of personal or family circumstances.

**Table 2:** Descriptive Statistics and Internal Consistency of the Study Variables (*N* = 342).

Variable	Mean	*SD*	Min	Max	Cronbach’s *α*
Decent Work Scale for Hospital Nurses (DWS-N)					
Total Decent Work	72.64	13.97	22	103	0.929
Meaningful and Fulfilling Work	17.52	3.70	5	25	0.886
Protection of Workers’ Rights	17.33	4.10	5	25	0.901
Supportive and Safe Working Environment	20.88	4.60	6	30	0.835
Appropriate Working Time and Work–Life Balance	9.09	2.81	3	15	0.842
Adequate Compensation	7.82	2.96	3	14	0.889
Work Engagement					
Overall Work Engagement	41.85	20.17	5	95	0.952
Burnout					
Personal Burnout	52.67	21.46	4	100	0.891
Work-Related Burnout	52.94	20.30	0	100	0.818
Client-Related Burnout	51.22	24.39	0	100	0.903
Job Crafting					
Increasing Structural Job Resources	14.31	4.11	5	25	0.895
Decreasing Hindering Job Demands	16.13	4.74	6	30	0.862
Increasing Social Job Resources	11.94	4.01	5	24	0.816
Increasing Challenging Job Demands	11.89	4.15	5	25	0.877

Note. *SD* = standard deviation; DWS-N = Decent Work Scale for Hospital Nurses.

**Table 3:** Correlations among Decent Work Dimensions, Work Engagement, Burnout Dimensions, and Job Crafting Dimensions (*N* = 342).

Variable	1	2	3	4	5	6	7	8	9	10	11	12	13	14
1. Meaningful and Fulfilling Work	—													
2. Protection of Workers’ Rights	0.547 ***		—											
3. Supportive and Safe Working Environment	0.477 ***	0.609 ***		—										
4. Appropriate Working Time and Work–Life Balance	0.519 ***	0.601 ***	0.439 ***	—										
5. Adequate Compensation	0.301 ***	0.407 ***	0.356 ***	0.548 ***	—									
6. Total Decent Work	0.750 ***	0.845 ***	0.797 ***	0.775 ***	0.638 ***	—								
7. Work Engagement	0.566 ***	0.381 ***	0.418 ***	0.416 ***	0.320 ***	0.550 ***	—							
8. Personal burnout	−0.477 ***	−0.340 ***	−0.314 ***	−0.535 ***	−0.340 ***	−0.509 ***	−0.381 ***	—						
9. Work-related burnout	−0.467 ***	−0.387 ***	−0.399 ***	−0.477 ***	−0.299 ***	−0.528 ***	−0.405 ***	0.722 ***	—					
10. Client-related burnout	−0.427 ***	−0.341 ***	−0.394 ***	−0.356 ***	−0.293 ***	−0.476 ***	−0.433 ***	0.532 ***	0.716 ***	—				
11. Increasing structural job resources	0.404 ***	0.144 **	0.213 ***	0.227 ***	0.137 *	0.294 ***	0.496 ***	−0.266 ***	−0.227 ***	−0.279 ***	—			
12. Decreasing hindering job demands	0.256 ***	0.094	0.167 **	0.241 ***	0.157 **	0.232 ***	0.167 **	−0.248 ***	−0.187 ***	−0.096	0.403 ***	—		
13. Increasing social job resources	0.177 **	0.124 *	0.152 **	0.201 ***	0.209 ***	0.218 ***	0.285 ***	−0.045	−0.058	−0.136 *	0.376 ***	0.384 ***	—	
14. Increasing challenging job demands	0.348 ***	0.148 **	0.177 **	0.214 ***	0.167 **	0.272 ***	0.453 ***	−0.175 **	−0.197 **	−0.227 ***	0.553 ***	0.274 ***	0.556 ***	—

Note. Values are Pearson’s correlation coefficients. * *p* < 0.05, ** *p* < 0.01, *** *p* < 0.001.

**Table 4:** Hierarchical Multiple Regression Analysis Predicting Work Engagement (*N* = 316).

Predictor	B	SE	*β*	95% CI for B		*p*	VIF
Step 1							
Age	0.159	0.112	0.080	[−0.061, 0.379]		0.157	1.005
Female (ref. Male)	−3.627	3.399	−0.060	[−10.314, 3.060]		0.287	1.005
Step 2							
Age	0.151	0.096	0.076	[−0.037, 0.339]		0.115	1.149
Female (ref. Male)	−1.836	2.721	−0.030	[−7.190, 3.518]		0.500	1.010
Meaningful and Fulfilling Work	2.427	0.322	0.435	[1.793, 3.061]		<0.001	1.651
Protection of Workers’ Rights	−0.136	0.333	−0.027	[−0.792, 0.520]		0.684	2.181
Supportive and Safe Working Environment	0.660	0.274	0.147	[0.121, 1.199]		0.017	1.834
Appropriate Working Time and Work–Life Balance	0.708	0.468	0.097	[−0.213, 1.629]		0.131	2.056
Adequate Compensation	0.670	0.368	0.098	[−0.054, 1.393]		0.070	1.446
Model statistics							
Model	*R* ^2^	Adjusted *R*^2^		Δ*R*^2^	F/ΔF		*p*
Step 1: Age + Female (ref. Male)	0.009	0.003		0.009	F(2, 313) = 1.477		0.230
Step 2: + five DWS-N dimensions	0.379	0.364		0.369	ΔF(5, 308) = 36.594		<0.001

Note. *N* = 316. B = unstandardized regression coefficient; SE = standard error; *β* = standardized regression coefficient; CI = confidence interval; VIF = variance inflation factor. Step 1 included age and sex, and Step 2 added the five DWS-N dimensions. Sex was dummy coded as female = 1 and male = 0; “Other” and “Prefer not to answer” were treated as missing. Regression analyses were conducted using complete cases for age and sex.

**Table 5:** Hierarchical Multiple Regression Analyses Predicting Burnout Dimensions (*N* = 316).

Predictor		B	SE		*β*	95% CI for B		*p*		VIF
Personal burnout										
Age		−0.122	0.102		−0.058	[−0.323, 0.080]		0.236		1.149
Female (ref. Male)		7.207	2.918		0.113	[1.465, 12.949]		0.014		1.010
Meaningful and Fulfilling Work		−1.730	0.346		−0.293	[−2.410, −1.050]		<0.001		1.651
Protection of Workers’ Rights		0.570	0.358		0.107	[−0.134, 1.273]		0.112		2.181
Supportive and Safe Working Environment		−0.101	0.294		−0.021	[−0.679, 0.477]		0.732		1.834
Appropriate Working Time and Work–Life Balance		−3.021	0.502		−0.393	[−4.008, −2.033]		<0.001		2.056
Adequate Compensation		−0.504	0.394		−0.070	[−1.280, 0.272]		0.202		1.446
Work-related burnout										
Age		−0.379	0.100		−0.189	[−0.575, −0.183]		<0.001		1.149
Female (ref. Male)		1.106	2.836		0.018	[−4.474, 6.686]		0.697		1.010
Meaningful and Fulfilling Work		−1.443	0.336		−0.256	[−2.104, −0.782]		<0.001		1.651
Protection of Workers’ Rights		0.014	0.348		0.003	[−0.670, 0.698]		0.967		2.181
Supportive and Safe Working Environment		−0.475	0.286		−0.104	[−1.037, 0.087]		0.097		1.834
Appropriate Working Time and Work–Life Balance		−2.187	0.488		−0.298	[−3.146, −1.227]		<0.001		2.056
Adequate Compensation		−0.201	0.383		−0.029	[−0.956, 0.553]		0.600		1.446
Client-related burnout										
Age		−0.306	0.126		−0.129	[−0.554, −0.059]		0.015		1.149
Female (ref. Male)		−3.614	3.577		−0.050	[−10.654, 3.425]		0.313		1.010
Meaningful and Fulfilling Work		−1.651	0.424		−0.246	[−2.485, −0.817]		<0.001		1.651
Protection of Workers’ Rights		0.068	0.438		0.011	[−0.795, 0.930]		0.878		2.181
Supportive and Safe Working Environment		−0.967	0.360		−0.179	[−1.676, −0.259]		0.008		1.834
Appropriate Working Time and Work–Life Balance		−0.894	0.615		−0.103	[−2.105, 0.316]		0.147		2.056
Adequate Compensation		−0.907	0.484		−0.111	[−1.858, 0.045]		0.062		1.446
Model statistics										
Outcome	Step 1 R^2^	Step 2 R^2^		Adjusted R^2^		ΔR^2^	ΔF (5, 308)		*p* for ΔF	
Personal burnout	0.022	0.361		0.346		0.339	32.642		<0.001	
Work-related burnout	0.027	0.338		0.323		0.311	28.969		<0.001	
Client-related burnout	0.020	0.254		0.237		0.234	19.288		<0.001	

Note. *N* = 316. B = unstandardized regression coefficient; SE = standard error; *β* = standardized regression coefficient; CI = confidence interval; VIF = variance inflation factor. Step 1 included age and sex, and Step 2 added the five DWS-N dimensions. Coefficients shown are from the final model (Step 2). Sex was dummy coded as female = 1 and male = 0; “Other” and “Prefer not to answer” were treated as missing. Regression analyses were conducted using complete cases for age and sex.

**Table 6:** Hierarchical Multiple Regression Analyses Predicting Job Crafting Dimensions (*N* = 316).

Predictor		B	SE		*β*	95% CI for B		*p*		VIF
Increasing structural job resources										
Age		0.002	0.022		0.005	[−0.042, 0.046]		0.930		1.149
Female (ref. Male)		−0.860	0.633		−0.070	[−2.106, 0.385]		0.175		1.010
Meaningful and Fulfilling Work		0.453	0.075		0.400	[0.306, 0.601]		<0.001		1.651
Protection of Workers’ Rights		−0.206	0.078		−0.202	[−0.359, −0.054]		0.008		2.181
Supportive and Safe Working Environment		0.077	0.064		0.085	[−0.048, 0.203]		0.226		1.834
Appropriate Working Time and Work–Life Balance		0.164	0.109		0.111	[−0.051, 0.378]		0.134		2.056
Adequate Compensation		0.023	0.086		0.017	[−0.145, 0.192]		0.784		1.446
Decreasing hindering job demands										
Age		−0.068	0.026		−0.148	[−0.119, −0.017]		0.010		1.149
Female (ref. Male)		−1.052	0.745		−0.075	[−2.518, 0.413]		0.159		1.010
Meaningful and Fulfilling Work		0.216	0.088		0.167	[0.043, 0.390]		0.015		1.651
Protection of Workers’ Rights		−0.306	0.091		−0.263	[−0.486, −0.127]		<0.001		2.181
Supportive and Safe Working Environment		0.153	0.075		0.146	[0.005, 0.300]		0.042		1.834
Appropriate Working Time and Work–Life Balance		0.388	0.128		0.230	[0.136, 0.640]		0.003		2.056
Adequate Compensation		0.059	0.101		0.037	[−0.139, 0.257]		0.561		1.446
Increasing social job resources										
Age		−0.089	0.023		−0.228	[−0.134, −0.045]		<0.001		1.149
Female (ref. Male)		−0.003	0.645		<−0.001	[−1.272, 1.266]		0.997		1.010
Meaningful and Fulfilling Work		0.062	0.076		0.056	[−0.088, 0.213]		0.415		1.651
Protection of Workers’ Rights		−0.172	0.079		−0.173	[−0.327, −0.016]		0.031		2.181
Supportive and Safe Working Environment		0.141	0.065		0.158	[0.013, 0.269]		0.031		1.834
Appropriate Working Time and Work–Life Balance		0.161	0.111		0.112	[−0.058, 0.379]		0.149		2.056
Adequate Compensation		0.171	0.087		0.127	[−0.001, 0.343]		0.051		1.446
Increasing challenging job demands										
Age		0.020	0.023		0.050	[−0.025, 0.065]		0.375		1.149
Female (ref. Male)		−0.633	0.651		−0.052	[−1.913, 0.647]		0.331		1.010
Meaningful and Fulfilling Work		0.376	0.077		0.332	[0.224, 0.527]		<0.001		1.651
Protection of Workers’ Rights		−0.139	0.080		−0.137	[−0.296, 0.017]		0.081		2.181
Supportive and Safe Working Environment		0.043	0.066		0.048	[−0.086, 0.172]		0.509		1.834
Appropriate Working Time and Work–Life Balance		0.111	0.112		0.076	[−0.109, 0.332]		0.320		2.056
Adequate Compensation		0.081	0.088		0.059	[−0.092, 0.254]		0.356		1.446
For compactness, the coefficients above are Step 2 (final model) coefficients. The hierarchical model statistics are:										
Outcome	Step 1 R^2^	Step 2 R^2^		Adjusted R^2^		ΔR^2^	ΔF (5, 308)		*p* for ΔF	
Increasing structural job resources	0.008	0.185		0.166		0.176	13.319		<0.001	
Decreasing hindering job demands	0.026	0.135		0.115		0.109	7.740		<0.001	
Increasing social job resources	0.041	0.107		0.087		0.066	4.571		<0.001	
Increasing challenging job demands	0.008	0.134		0.115		0.127	9.011		<0.001	

Note. *N* = 316. B = unstandardized regression coefficient; SE = standard error; *β* = standardized regression coefficient; CI = confidence interval; VIF = variance inflation factor. Step 1 included age and sex, and Step 2 added the five DWS-N dimensions. Coefficients shown are from the final model (Step 2). Sex was dummy coded as female = 1 and male = 0; “Other” and “Prefer not to answer” were treated as missing. Regression analyses were conducted using complete cases for age and sex.

## Data Availability

The data presented in this study are available from the corresponding author upon reasonable request. The data are not publicly available because they contain information that could compromise participant confidentiality.

## Public Involvement Statement

No public involvement in any aspect of this research.

## Guidelines and Standards Statement

This manuscript was prepared with reference to the Strengthening the Reporting of Observational Studies in Epidemiology (STROBE) Statement for cross-sectional studies [29].
